# Effects of xylem embolism on the winter survival of *Abies veitchii* shoots in an upper subalpine region of central Japan

**DOI:** 10.1038/s41598-020-62651-2

**Published:** 2020-04-20

**Authors:** Emiko Maruta, Mitsumasa Kubota, Takefumi Ikeda

**Affiliations:** 1 Department of Biology, Faculty of Science, Toho University, 2-2-1 Miyama, Funabashi Chiba, 274-8520 Japan; 2 Department of Bioresource Science, Faculty of Agriculture, Shizuoka University, 836 Oya, Surugaku Shizuoka, 422-8529 Japan; 3 Department of Forest Science, Graduate School of Life and Environmental Sciences, Kyoto Prefectural University, 1-5 Shimogamohangi-cho, Sakyo-ku Kyoto, 606-8522 Japan; 4 Present Address: Department of Biological Science, Faculty of Science, Kanagawa University, 2946 Tsuchiya, Hiratsuka, Kanagawa 259-1293 Japan; 5 Present Address: Daitou Techuno Green Inc., 1-2-3 Haramachida, Machida Tokyo, 194-0013 Japan

**Keywords:** Ecophysiology, Forest ecology

## Abstract

At high elevations, winter climatic conditions frequently cause excessive drought stress, which can induce embolism in conifer trees. We investigated the formation and repair of winter embolism in subalpine fir (*Abies veitchii*) growing near the timberline. We found a complete loss in xylem conductivity [100% percent loss of conductivity (PLC)] at the wind-exposed site (W+) and 40% PLC at the wind-protected site (W−). A PLC of 100% was far above the embolism rate expected from the drought-induced vulnerability analysis in the laboratory. At the W+ site, a PLC of 100% was maintained until May; this suddenly decreased to a negligible value in June, whereas the recovery at the W− site started in late winter and proceeded stepwise. The contrast between the two sites may have occurred because of the different underlying mechanisms of winter embolism. If most tracheids in the xylem of 100% PLC are air-filled, it will be difficult to refill quickly. However, embolism caused by pit aspiration could be restored rapidly, because aspirated pits isolate tracheids from each other and prevent the spread of cavitation. Although severe embolism may cause frost damage of needles, it may have a role in holding water within the stem.

## Introduction

During winters, trees growing at high altitudes or latitudes are often exposed to conditions that are unfavourable to the plant’s water status^1^. Water uptake is blocked by the frozen soil and stem for months, whereas high-speed winds and intense radiation can cause water loss from trees, leading to a condition known as “frost drought”^2^. In conditions of frost drought, evergreen conifers are thought to prevent winter desiccation of needles by closing stomata and increasing cuticle thickness^2,3^. However, during winter, the cuticles frequently fail to prevent water loss, resulting in desiccation-related damage during winter, and this is considered the primary cause of the krummholz growth form^4,5^. Thus, winter desiccation could determine the altitude limit of the krummholz^2^, even if it may not generally explain the cause of treeline formation^6^. Low cuticular resistance to water loss is thought to result from inadequate cuticle development over short and cool subalpine summers^1^, cuticle abrasion by wind-blown ice particles^4,5^ and a lack of epicuticular wax^5,7^.

If shoot tips can be replenished with water stored in tree stems during winter, conifer trees could avoid desiccation damage at high elevation. Several studies have reported that water stored in the living tissues of the stem could be transported through the xylem conduits to replenish water depletion at shoot tips when soil water is frozen and unavailable to trees during winter, such as in *Pinus sylvestris* in boreal forests^8^, *Picea abies* in the European Central Alps timberline^9^, and *Larix kaempheri* at the timberline of Mt. Fuji^10^. Even in mid-winter, water can move from stems to twigs in sun-exposed and thawed parts of trees on calm and sunny days^9,10^. However, water supply from the stem can often be impaired because of winter embolism. For instance, limited water supply due to xylem embolism induced severe frost damage in *L. kaempheri* trees at the timberline^10^.

Frost drought can often reduce the water potential (ψ) of shoots to the vulnerability threshold for the onset of drought-induced embolism caused by the entrapment of air in xylem conduits^11^. Upon freezing, vulnerability to drought-induced embolism could increase due to low apoplastic water potential over ice front^12,13^. Additionally, trees in cold habitats can often be subjected to freeze–thaw events during winter. As water in the xylem freezes, gases dissolved in water form bubbles that expand during subsequent thawing under the low water potential of xylem sap and consequently result in embolism^11,14^. As larger elements comprise higher amounts of dissolved gas, larger bubbles form within the ice, thereby increasing the risk of embolism. Accordingly, the vulnerability to freeze–thaw-induced embolism positively correlates with the conduit diameter^14–17^. Thus, the altitude limits of angiosperm tree species with large diameter-conduits are reportedly associated with their vulnerability for freeze–thaw-induced embolism^17,18^. Thus, conifers with smaller conduits are reportedly more resistant to freeze–thaw-induced embolism than angiosperm tree species^17,19–22^. This phenomenon provides an explanation for the dominance of conifers at high altitudes and latitudes^21^.

At alpine timberlines, however, the occurrence of xylem embolism during the winter months has frequently been demonstrated in conifers^23–25^. This occurs because of excessive drought stress in combination with multiple freeze–thaw events^9^. Conifers growing at high elevation were observed to exhibit a wide range of winter embolism: from moderate [25–60% percent loss of conductivity (PLC)]^9,23,24,26,27^ to high (almost 100% PLC)^24,25^. Recovery from moderate embolism has been reported to start in late winter, and complete recovery of xylem conductivity occurs by the time the vegetative growth period begins^26^. In contrast, at the uppermost timberline ecotone, severe embolism up to 100% did not recover in late winter^24^. Extensive embolism during winter will have harmful effects on photosynthesis and subsequent growth in the following winter, if refill is delayed or failed^12^.

In the- present study, we monitored the intensity of winter desiccation, formation and repair of winter embolism, and water balance of the stem using a dendrometer in subalpine fir (*Abies veitchii*) growing near the timberline. A comparison of wind-exposed and wind-protected *A. veitchii* trees could allow us to compare different degrees of winter embolisms. Our aim was to determine (1) whether the extent to which embolism depended on the severity of winter in the habitats, (2) whether the extent of embolism correlated with the timing of embolism formation and repair and (3) whether heavy embolism caused inadequate water balance and was lethal to shoot tips of conifers. Thus, the causes and consequences of winter embolism would be inferred by comparing *A. veitchii* trees at each site.

## Material and methods

### Study site and plant material

Studies were performed on a ridge (36°4′N, 138°20′E, 2,226 m above sea level) of the upper subalpine region of the northern Yatsugatake Mountains in central Japan during the winter of 2006–2007. This site experiences strong westerly winds, especially during the winter months. The ground surface remains covered with snow from early December to late May with a maximum depth of 1 m in mid-winter.

**Figure 1:**
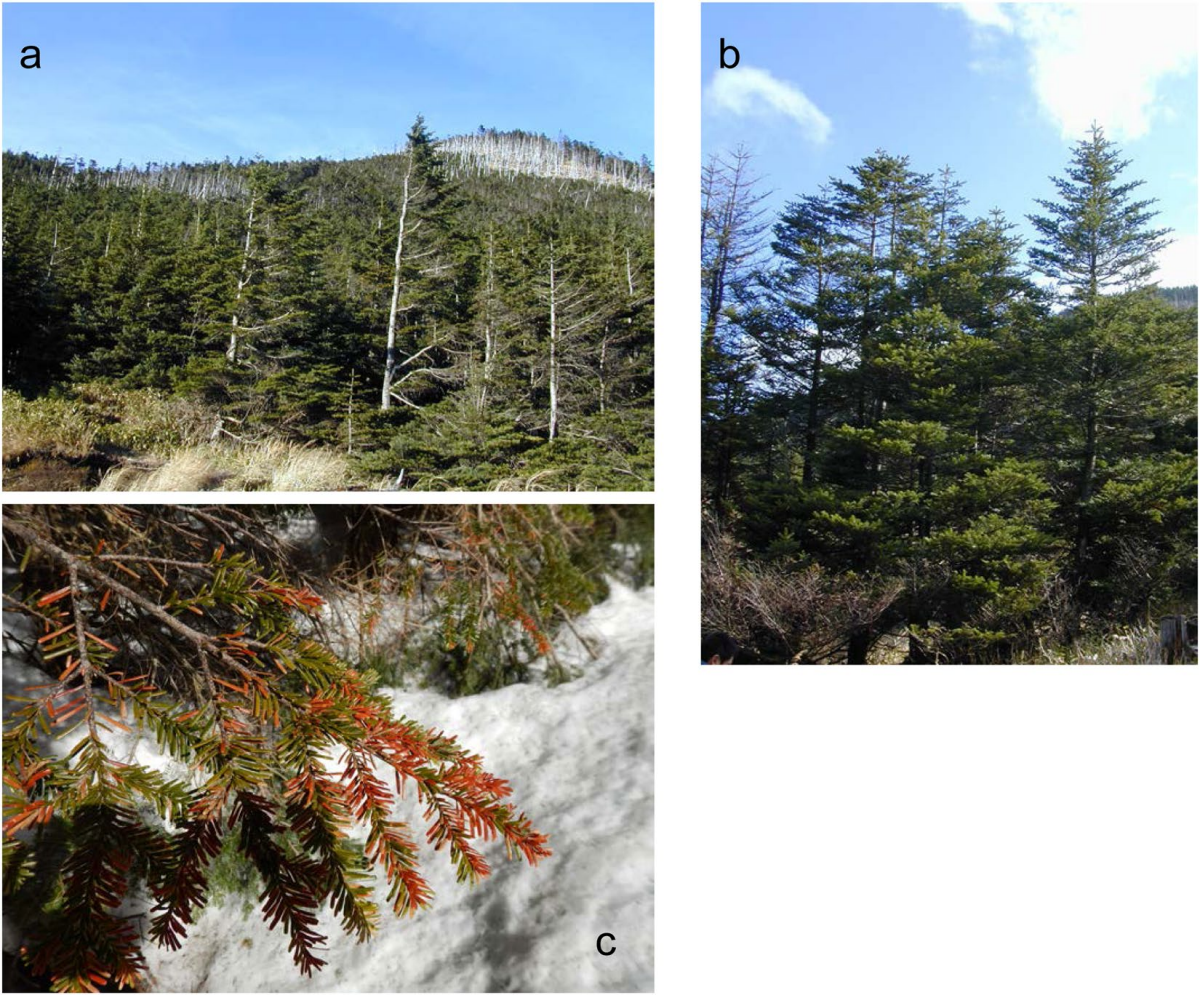
Tree forms of *Abies veitchii* at the study site (2,226 m above sea level) on the northern Yatsugatake Mountains in central Japan. (**a**) Flagged crown trees at the wind-exposed site (W+) of a pass. (**b**) Normal trees at the wind-protected site (W−). (**c**) Brown coloured needles of *A. veitchii* trees at the W+ site in late March. These needles were shed by late June.

The tree forms of the subalpine fir, *A. veitchii*, vary according to wind exposure, which is influenced by the microtopography of the region. The winter water relations of flagged crown trees [7 cm diameter breast height (DBH), 4 m high] at the most wind-exposed site (W+) were compared with those of normal crown trees (18 cm DBH, 7 m high) at the wind-protected site (W−); the W+ and W− sites were separated by a distance of 300 m (Fig. 1a,b). The needle longevity of the windward shoots was 3 years for W+ trees and 9 years for W− trees. In the period from late winter to early spring, shoot dieback was observed in trees at the W+ site (Fig. 1c), but not in the trees at the W− site. These observations suggest that shoot dieback, which occurs each winter, may be responsible for the shorter longevity of needles on the trees at the W+ site and, consequently, for the formation of flagged trees.

### Temperature measurements

Air temperature was measured every 30 min at a height of 2 m using a Pt-100 sensor (HMP155, Vaisala Corp., Helsinki, Finland) at the W+ site. The temperatures of the xylem (T_x_) of *A. veitchii* (wind-exposed and sun-lit branches and stems at a height of 1.5–2 m) and soil (T_0_, at the soil surface and T_20_, at a depth of 20 cm) were measured every 30 min using 0.3 mm thermocouples and recorded with a datalogger (Model CR10, Campbell Scientific Corp., Utah, USA). To measure the xylem temperature, thermocouples were inserted to a depth of 0.8 cm into the xylem of branches (2 cm diameter on a W+ tree and 5 cm diameter on a W− tree) and to a depth of 1.5 cm into the xylem of stems (7 cm diameter on a W+ tree and 18 cm diameter on a W− tree).

### Sampling

To monitor water relations over the winter season, westward and sun-lit branches were harvested from each of the four *A. veitchii* trees at both W+ and W− sites. The trees were different from those used for temperature measurements. Samples were collected just before sunrise only in October, May and June; whereas they were collected at noon during the winter (December–April). In the winter sample collection for pre-dawn water status observations was not necessary as stomata remained closed. The samples were immediately sealed and placed in a plastic bag kept below 5 °C and transported to the laboratory.

### Cuticular resistance, relative water content and needle viability

The cuticular resistance to water loss (r_c_), the relative water content (RWC), and viability of needles were measured as previously described by Hadley and Smith^4,5^ with slight modifications. Shoots (n = 6) were rehydrated by placing the cut end in water at room temperature (17 °C ± 2 °C) for 24 h. The shoots were then cut into the 1-, 2-, and 3-year-old portions and the cut end was sealed with paraffin. Under dark conditions, the rate of water vapour loss from needles was gravimetrically determined at regular intervals of 1 h until the rate became constant^7^. Cuticular resistance r_c_ (s^−1^m) was calculated using an analogy of Ohm’s Law for electrical circuits as follows:

1 $${I}_{v}=({e}_{s}-{e}_{a})/{r}_{c}$$

where J_v_ (g m^−2^ s^−1^) is the rate of water vapour loss from needles, e_s_ (g m^−3^) is the water vapour concentration inside a needle (assuming saturation at needle temperature), and e_a_ is the water vapour concentration in air. The measured r_c_ is certainly not the needle resistance but the cuticular resistance because stomata will be closed under dark conditions.

The needle RWC (%) of 2-year-old segments (n = 4) was determined from the measurements of fresh (FW) and dry weight (DW) as follows:

2 $${\rm{RWC}}=({\rm{FW}}-DW)/(TW-DW)\times 100$$

where TW is the turgid needle weight after soaking the needles in distilled water for 3 days at 5 °C. The DW of needles was measured after drying the needles at 80 °C for 48 h.

To measure needle viability, needles were collected from 3-year-old shoots (n = 20), and the percentages of needles with >50% green surface area were calculated.

### Water potential (ψ)

The water potential (ψ) was determined for approximately 10 cm long tips of 5-year-old shoots (n = 3–5) using a pressure chamber (Model 3000, Soil moisture Equipment Corp., Santa Barbara, CA, USA).

### Xylem conductivity

Xylem hydraulic conductivity was measured with modified apparatus as described previously^28^. For xylem conductivity measurements, 12 cm-long (5-year-old) segments (n = 4) were cut from branches under water in the laboratory. While under water, approximately 5-mm wide bark ends were removed. Subsequently, the segments were fitted to the conductivity system connected to a reservoir with distilled and filtered (0.22 μm) water and the initial hydraulic conductance was measured at a pressure difference of 0.01 MPa. The rate of embolism, described as percent loss of conductivity (PLC), was calculated from the ratio of initial (K) to maximal conductivity (K_max_) of xylem after 10 min flushing (0.5 MPa), according to the following equation:

3 $$PLC=(1-K/{K}_{max})\times 100$$

### Vulnerability analysis

Vulnerability curves were obtained using the air injection method as described previously^29^. Samples were obtained in December, in addition to the samples obtained monthly. Branches (5-year-old, 12 cm in length, and 8–15 mm in diameter, n = 4) were cut under water and placed in a double-ended pressure chamber with both ends protruding. One end of each segment was attached to the conductivity system. Initial xylem hydraulic conductance was measured as described for the xylem conductivity measurements above. Subsequently, the air pressure was progressively increased until the hydraulic conductivity readings were 0. A vulnerability curve was constructed for each segment by plotting PLC values against the negative air injection pressure. A Weibull curve^30^ were fit using a nonlinear procedure (SAS Institute Inc.) and a 50 percent loss of conductivity (P50) was estimated.

### Safranin staining of stem

To identify functional xylem areas, fresh 2-year-old stem segments (n = 3) were cut under water and connected to a reservoir with 0.1% (w/v) aqueous safranin under a pressure of 0.01 MPa.

### Dendrometer measurements

Variations in the diameter of branches and stems were continuously measured using point dendrometers (Type DD, Type DR, Ecomatik Corp., Munich, Germany). Three trees were selected at each site (W+ and W−) for dendrometer measurements. Each branch and stem of these trees was equipped, on their west side, with a point dendrometer at heights of 1.5 m and 2 m at the W+ and W− sites, respectively. Sensing rods of the dendrometers were held with a constant force against the smoothed bark surface of the branches (diameters of 2.2 cm and 5.0 cm at the W+ and W− sites, respectively) and the stems (diameters of 7 cm and 18 cm at the W+ and W− sites, respectively). The resolution of the dendrometers was 1.5 μm and the temperature coefficient of the sensor was less than 0.2 μm. According to the manufacturer, these dendrometers are functional at temperatures as low as −30 °C. Data were collected at 30-min intervals using a CR10X datalogger.

### Statistical analysis

The statistical significance of the differences in various measurements between the W+ and W− sites was determined using the Student’s *t*-test at the 5% probability level. One-way analysis of variance was used to detect significant differences among different shoot ages and between the W+ and W− sites.

## Results

### Microclimate

**Figure 2:**
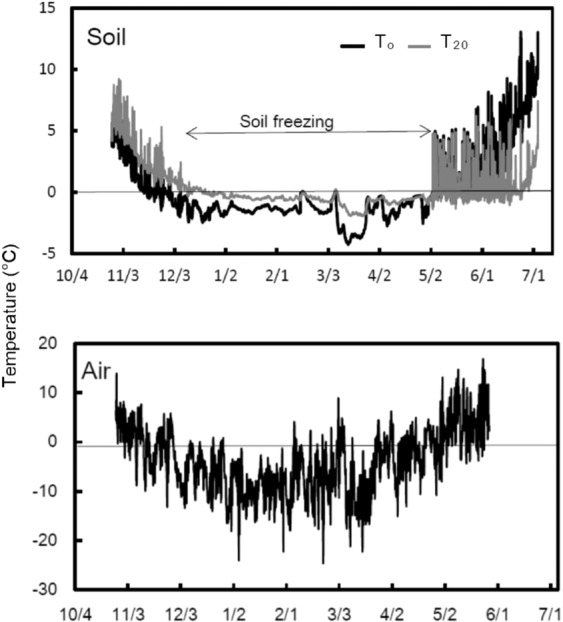
Seasonal variations in soil and air temperatures from late-October to June at the W+ site. Soil temperatures were measured at the soil surface (T_0_) and at a depth of 20 cm (T_20_).

The soil (T_0_ and T_20_) remained frozen from December to April (Fig. 2). Snow cover remained during this period with a maximum depth of 1 m. The air temperature remained mainly below 0 °C throughout the winter, and was occasionally above 0 °C. January was the coldest month; air temperature in January consistently remained below 0 °C, with the lowest measurement reaching −25 °C.

### Winter needle desiccation and death

**Figure 3:**
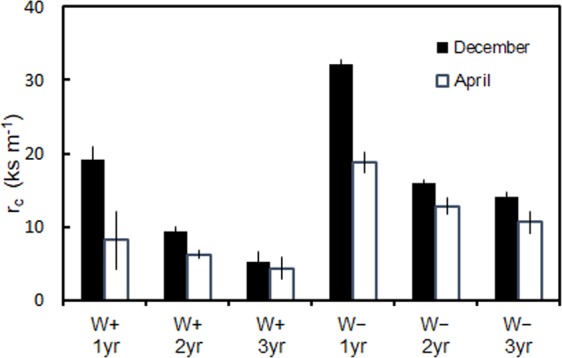
Cuticular resistance (r_c_) of 1-, 2-, and 3-year-old needles of *A. veitchii* trees collected from the W+ and W− sites in mid-December and early April. Mean ± SE.

The r_c_ of needles was significantly lower at the W+ site than that at the W− site within each age group (1-, 2-, and 3-year-old shoots) during both December and April (P < 0.05) (Fig. 3). At both sites, the r_c_ of 1-year-old needles was the highest and decreased with the increasing age of needles in both December and April. The r_c_ of needles of all ages decreased from December to April at both sites. This decrease in r_c_ was the greatest for 1-year-old needles at both sites, reducing to nearly half in April in comparison to that in December; however, the decline in the r_c_ of 2- and 3-year-old needles from December to April was relatively small or not significant (P < 0.05) at both sites. These data indicate that the decline in r_c_ is the greatest during the first winter and subsequently lower in the succeeding winters.

**Figure 4:**
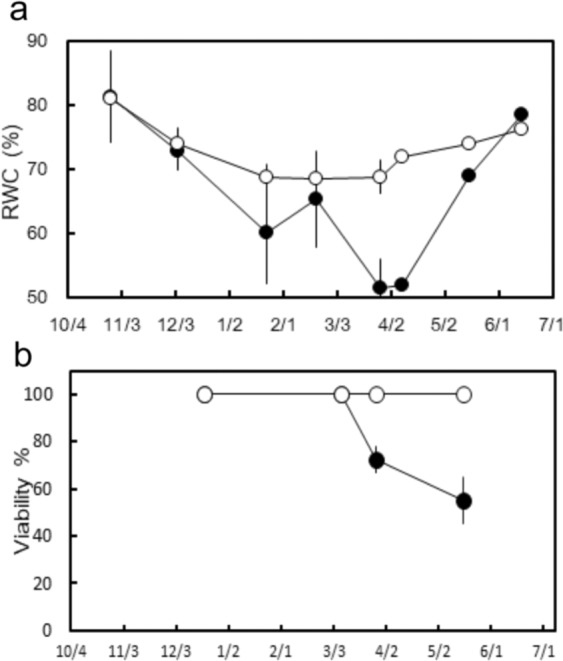
(**a**) Seasonal variations in the relative water content (RWC) of 2-year-old needles collected from late-October to June, (**b**) seasonal variations in the needle viability of needles collected from December to May. W+ site (closed circles) and W− site (open circles). Mean ± SE.

Needles from both sites retained a high RWC of more than 70% until early December (Fig. 4a). In the period from January to April, the RWC of needles from the W+ site was significantly lower than that of needles from the W− site (P < 0.05), possibly due to the low r_c_. From January, the RWC of needles collected from the W− site plateaued and stayed nearly constant at a safe level of 68% for the remainder of the winter, whereas the RWC of needles collected from the W+ site decreased to a lethal level of 50% in late March. By mid-May, needles that survived were green and their RWC had recovered to nearly 70%, whereas those that did not survive the winter were brownish in colour and showed the lowest level of RWC in mid-May (data not shown).

Although needle death was not observed at both sites by mid-winter, some needles from wind-exposed shoots at the W+ site turned brown in late March (Fig. 1c). Needle viability at the W+ site declined to approximately 70% by late March and further to approximately 50% in mid-May, whereas no needle death was observed at the W− site (Fig. 4b). Thus, needle death at the W+ site coincided with a steep decline in RWC in late March.

### Seasonal course of water potential (ψ), xylem embolism (PLC), and refilling in spring

**Figure 5:**
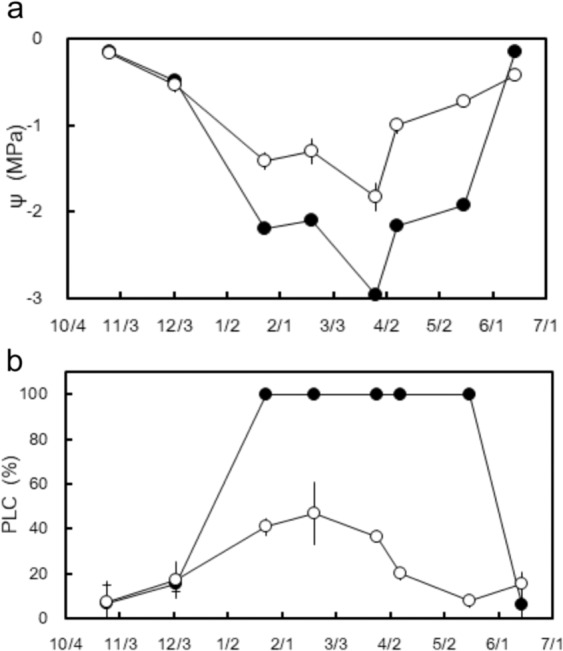
(**a**) Seasonal variations in shoot water potential (ψ), (**b**) seasonal variations in embolism, expressed as percent loss of conductivity (PLC) collected from late-October to June. W+ site (closed circles) and W− site (open circles). Mean ± SE,.

Water potential (ψ) exhibited a similar trend to the RWC of needles over the course of the winter. In November and December, the ψ was the same between the W+ and W− sites, with a value higher than −0.5 MPa (Fig. 5a). The ψ of shoots from both sites declined by mid-January and remained low for the remaining winter months. The ψ of shoots from the W+ site was significantly lower than that of shoots from the W− site (*P* < 0.05) (lowest values on March 26, 2007: −3 MPa for W+ and −1.8 MPa for W−). For a short period of 13 days from March 26 to April 8, the ψ increased approximately 0.8 MPa in shoots from both sites. Then, the ψ of shoots from both sites gradually increased to the highest level of more than −0.5 MPa on June 14.

In early winter (November and December), PLCs were below 20% for shoots from both sites (Fig. 5b). From January to March, the PLC for shoots from the W− site remained constant at approximately 40% and subsequently started declining in April. In May, the PLC was negligible. By contrast, the PLC for shoots from the W+ site increased abruptly from a negligible level in December to 100% in January, which was maintained until May, indicating complete embolism. Subsequently, the PLC of shoots from the W+ site recovered rapidly, reaching a value of <10% in June.

**Figure 6:**
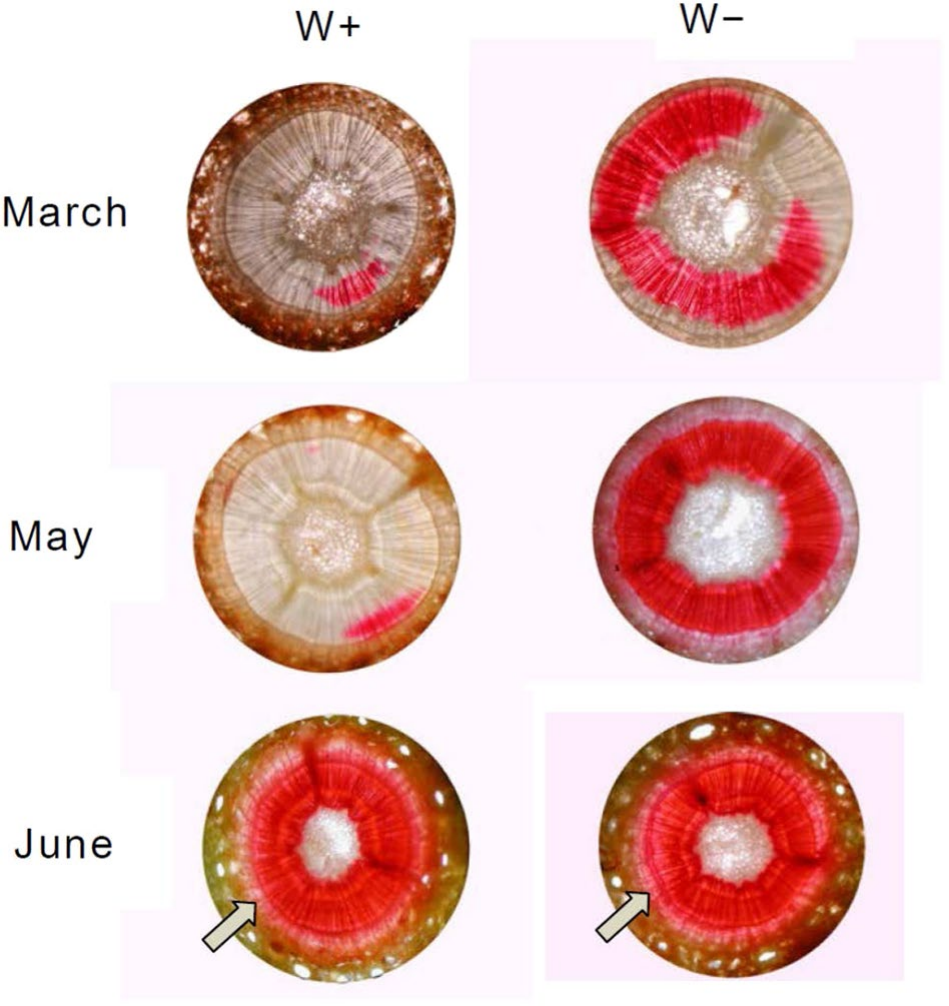
Safranin staining of xylem in the branches of *A. veitchii* harvested from the W+ and W− sites from March to June. Images show cross sections of branches. Area stained red with safranin indicates hydraulically intact regions, whereas unstained areas represent embolised regions. Arrows indicate newly formed xylems.

Dye staining experiments with twigs revealed that nearly 100% of the xylem area in shoots from the W+ site was unstained in March and May, indicating complete embolism blocked the flow of staining solution (Fig. 6). By contrast, only 25% of the xylem area was unstained in shoots from the W− site in March. Subsequently, all portions of the xylem were stained for shoots from the W− and W+ sites in May and June, respectively. Thus, the unstained area of embolised xylem and timing of complete refilling demonstrated by the dye staining experiments (Fig. 6) coincided with the seasonal change in PLC (Fig. 5b). Newly formed annual xylems were found in July.

### Vulnerability curves

**Figure 7:**
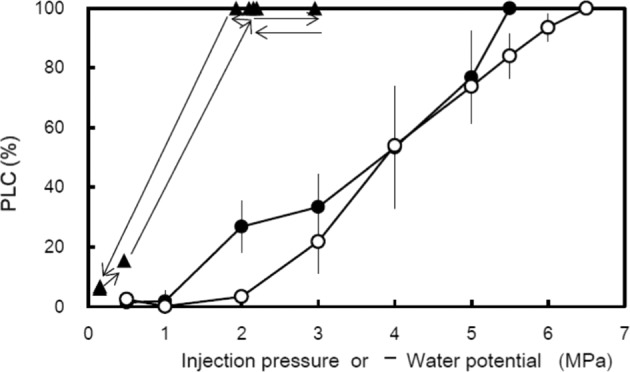
Vulnerability curves showing PLC in xylem versus air pressure used in air-injection experiments for shoots collected from the W+(closed circles) and W−(open circles) sites in December. Data represented as mean ± SEM (n = 4). PLC versus water potential (ψ) from native data (W+ site, late-October, 2006 to June, 2007) shown in Fig. 5 are indicated with triangles.

Vulnerability curves obtained using the air injection method showed that shoots from both sites exhibited almost the same vulnerability thresholds (Fig. 7). The 50% vulnerability values(P50) were −3.52 ± 0.53 MPa and −4.02 ± 0.80 MPa for shoots from the W+ and the W− sites, respectively. There was no significant difference in the P50 values between both sites (P > 0.05). The PLC of 100% found in the field data of the W+ site corresponded with the water potentials of −2 to −3 MPa at which PLCs were less than 40% in the vulnerability analysis (Fig. 7).

### Variation in dendrometer measurements of branch and stem diameter

**Figure 8:**
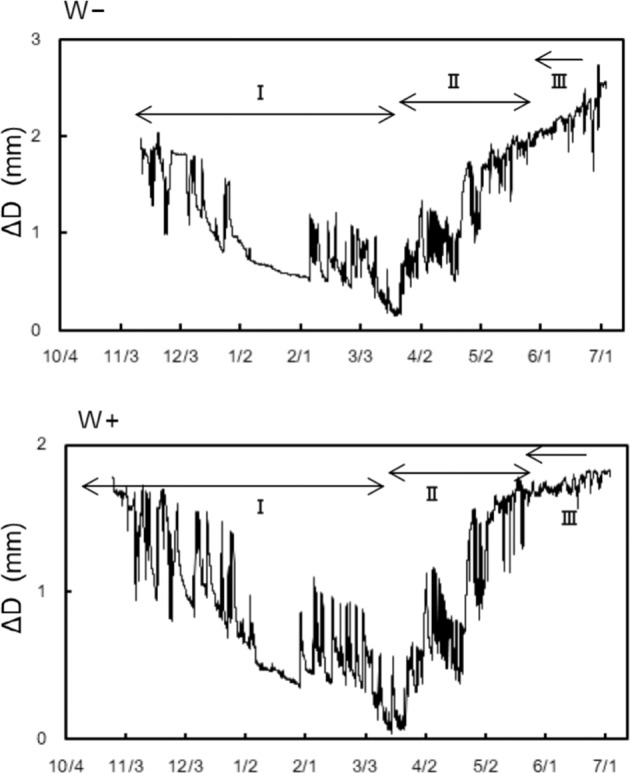
Seasonal variations in diameter (ΔD) for representative branches at the W+ and W− sites. This variation is divided into three distinct phases: phase I indicates winter shrinkage, phase II indicates spring swelling, and phase III indicates diameter increment. The values of ΔD are adjusted relative to the lowest value, which is considered as zero. Variations in stem diameter during winter followed the same trend as that of branches and therefore are not shown.

In December and January, the major trend in diameter change was a simple shrinkage (Fig. 8), which was consistent with sub-zero temperatures (Fig. 2). Conversely, in February and March, short-term fluctuations associated with shrinkage and swelling were observed regularly (Fig. 8), which were consistent with freeze-thaw cycles (Fig. 2). A detailed explanation is presented in Supplementary Fig. S2. The time series showed an inflex point in late March, following which the branch diameter increased progressively until late May (Fig. 8). After reaching the same level as that observed in the beginning of measurements, the increase in diameter was sustained potentially after mid-June due to wood formation, consistent with the results of safranin staining experiments (Fig. 6).

## Discussion

In this study, *A. veitchii* trees overwintering at the W+ site experienced severe frost drought effects, such as needle desiccation due to insufficient cuticular protection and consequent needle mortality in late winter (Figs. 3 and 4), which were in agreement with severe frost drought effects found in several timberline conifers of previous reports^2,4,5^. Despite the W− site being only 300 m apart and being exposed to an identical climate, except for wind exposure, the frost drought was milder and no mortality was observed. Winter embolism also differed between the two sites (Fig. 5b). At the W− site, embolism formation and repair progressed gradually, with a maximum embolism rate of 40%. At the W+ site, the PLC suddenly increased from a negligible value in December to 100% in January, maintaining a 100% PLC until May, followed by a sudden decrease to a low value in June. The contrast between the two sites may have occurred because of the different underlying mechanisms causing embolism.

The 100% PLC observed throughout the winter at the W+ site was far above the embolism rate expected from the drought-induced vulnerability curve established in the laboratory^9^ (Fig. 7). This discrepancy between the field study and the experiments reflecting only drought-induced embolism may be caused by another factor, such as repeated freeze–thaw events^9^. Mayr *et al*.^31,32^ hypothesised that such winter embolism can be induced by a combination of excessive drought stress and multiple freeze–thaw events, a common phenomenon in trees growing at high elevations. However, from December 4 to January 23, the xylem of branches at the W+ site may have experienced only five freeze–thaw events (Figs. 2 and 8). Therefore, it is unlikely that the only combination of low ψ and small number of freeze–thaw cycles would cause the formation of gas bubbles to lead to complete conductivity loss in xylems of W+ twigs in the present study. Additionally, if most tracheids in xylems of 100% PLC were air-filled as a result of multiple freeze–thaw events, it should be difficult to refill in xylems that are completely air-filled. Thus, it is hypothesised that the valve-like closing mechanism of torus–margo pits^19,33^ may be primarily responsible for the complete loss of xylem conductivity at the W+ site. Hammel^19^ suggested that, upon freezing, pits of conifers may be aspirated and tracheids may be isolated from each other, thereby preventing widespread xylem cavitation. Pit aspiration may be an adaptation to severe winters for conifers overwintering at high elevation because pit aspiration can prevent the spread of cavitation and allow complete recovery of xylem conductivity. However, pit aspiration may cause a trade-off between preventing the spread of cavitation and impairing xylem conductivity.

The underlying mechanism involved in the repair of embolism may also be different among both the sites. Recovery from embolism at the W− site started in the late winter and proceeded stepwise for several weeks (Fig. 5b), in agreement with the recovery process reported by Mayr *et al*.^26^. Recovery from winter embolism is thought to be based on both passive and active processes^26^. Snow melting water on branches could shift to air-filled tracheids via intact xylem, phloem, and parenchyma and can be used as a source of water increasing ψ^26^. In the present study, the refilling process at the W− site was associated with uptake of soil water when the soil temporarily melted due to rainfall in the late winter (Supplemental Fig. S3). Then, active, cellular processes such as the increase in osmotically active sugars and aquaporin, may play a role in refilling xylem even under winter conditions^26,34^. By contrast, recovery from embolism at the W+ site did not occur until June (Fig. 5b), nevertheless, the soil was completely thawed by early May (Fig. 2). A similar phenomenon has been shown in *P. abies* growing at the timberline of the Central Alps^24^. Due to complete loss of conductivity, the embolized xylem could not be supplied with water to refill. In addition, aspirated torus of pit could not return to the normal position, which may have caused continued embolism. Based on safranin staining experiments (Fig. 6) and dendrometer data (Fig. 8), new annual xylem was found on June 14, when a recovery from embolism was demonstrated at the W+ site, indicating that formation of a new annual xylem may act as a trigger for pit opening.

The needle RWC at the W− site decreased markedly during early winter, but it remained constant during the remainder of the winter season (Fig. 4a). Conversely, the needle RWC at the W+ site decreased to a lethal level by late March (Fig. 4a). The difference in the needle RWC between the two sites may reflect the difference in water balance of shoot tips during winter.

Reversible stem diameter changes as measured using a dendrometer reflect water dynamics of trees under frozen soil^8,35–38^. Water in the living tissues (xylem parenchyma, phloem, and cambium) of the stem is withdrawn from the cells into apoplastic space of xylem due to extracellular freezing. The stem diameter shrinks during freezing depending on the difference between the expansion of apoplastic ice and freezing shrinkage of living tissue^39^. Thus, no mass flow of water through xylem conduits can occur as long as the stem diameter shrinks^9,27,39^. Shrinking could continue for months in winter, because the volume fraction of xylem apoplast is much larger than that of water in living tissues that can be frozen extracellularly^8^. During thawing in freeze-thaw cycle of the diameter change, water would return to the living cells from apoplast and be available for water supply of the distal tissues^35^.

In December and January, decreases in needle RWC at the W− site may have resulted from the cessation of water supply from the stem when “shrinkage” was the dominant pattern of the diameter change in accordance with the sub-zero temperatures (Fig. 8, Supplemental Fig. S2 for details). Beginning in early February, the freeze–thaw cycle frequently occurred with daytime temperatures above 0 °C (Fig. 8, Supplemental Fig. S2 for details). Shoots at the W− site maintained a constant level of needle RWC until early April (Fig. 4b) as water in the stem might be thawed and be transferred to the tip needles during the day, which is consistent with the findings of previous studies^8–10^. Conversely, the needle RWC at the W+ site continued to decrease until early April (Fig. 4b) as severe embolism blocked water supply of shoot tips even when melting. These results indicate that water transport from the stem to shoot tips is necessary to maintain the water content of needles above the lethal level throughout the winter, implying that severe embolism is primarily responsible for needle desiccation in cold habitats.

Desiccation injury of needles due to heavy embolism may cause hydraulic segmentation between the stem and tip needles^40,41^, which can protect stem water from dissipation despite leading to krummholz formation. Experiments should be conducted to observe the pit behaviour using cryo-SEM and to reveal the cause and mechanism that lead to heavy winter embolism in conifers growing at W+ sites at high elevations.

## Supplementary information

Supplementary information.
