# Supplementary material for: Effects of xylem embolism on the winter survival of Abies veitchii shoots in an upper subalpine region of central Japan
Source: Sci Rep. 2020 Apr 20;10:6594. doi: 10.1038/s41598-020-62651-2 (PMC7171099; doi:10.1038/s41598-020-62651-2)
Supplement: Supplementary file 1 — Supplementary information. [file 41598_2020_62651_MOESM1_ESM.pdf]

Effects of xylem embolism on the winter survival of *Abies veitchii* shoots in an upper subalpine region of central Japan

Authors

Emiko Maruta, Mitsumasa Kubota, Takefumi Ikeda

Supplementary figures:

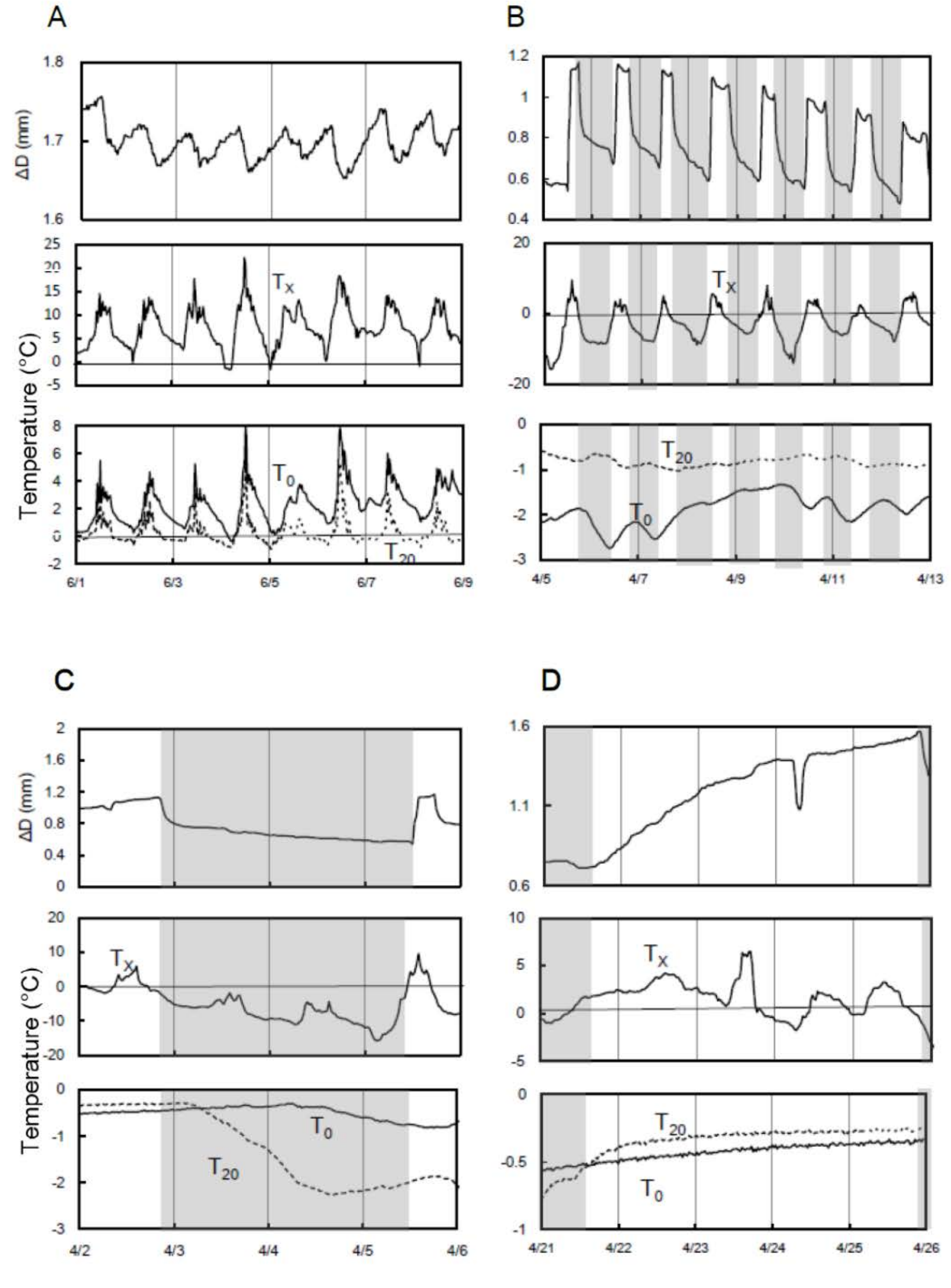

Fig. S1 Typical patterns of the variation in branch diameter (ΔD) during (A) transpiration cycle, (B) freeze-thaw cycle, (C) freezing period, and (D) rehydration period for a tree at the W+ site. The corresponding soil (T_0 and T_{20}) and branch (T_x) temperatures are shown. Grey shading delineates a phase of decrease in diameter due to freezing. (A) Transpiration cycle: Diurnal changes in diameter, shrinkage in daytime and swelling in nighttime reflect changes in the amount of reservoir water as induced by transpiration[36]; (B) Freeze-thaw cycle: Diurnal changes in diameter, swelling in daytime and shrinkage in nighttime oppositing to those of transpiration cycle reflect changes in the amount of reservoir water in accordance with freezing and thawing in the apoplast. The diurnal amplitude of the change in freeze-thaw cycles (B) is 5–10 times larger than that in the transpiration cycle (A); (C) Freezing period: Ice nucleation in the apoplast creates a high water driving force from living tissues to the apoplast because water potential of ice depends on temperature; water potential of ice by decreases 1.22 MPa per 1°C in temperature[40]. The diameter continues to shrink in accordance with the declining level of water stored in reservoirs depending on ice temperature. (D) Rehydration period: When soil water is occasionally available after rainfall, the diameter swells due to the rehydration of reservoirs (see Figs S3 and S4 for details).

Fig. S2

A

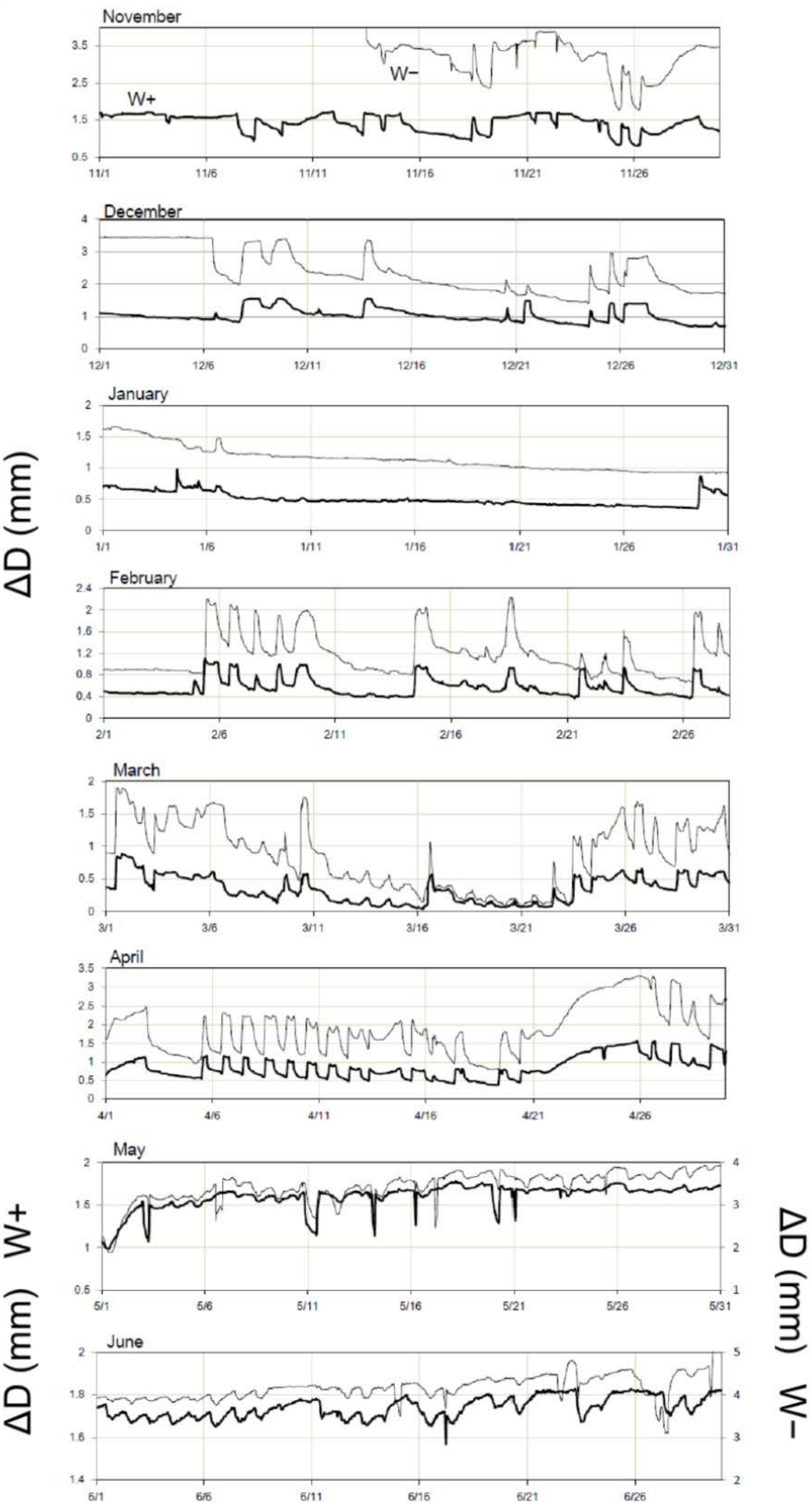
November
W−
W+
December
January
February
March
April
May
June
ΔD (mm)
ΔD (mm) W+
ΔD (mm) W−

Fig.S2 (Continued)

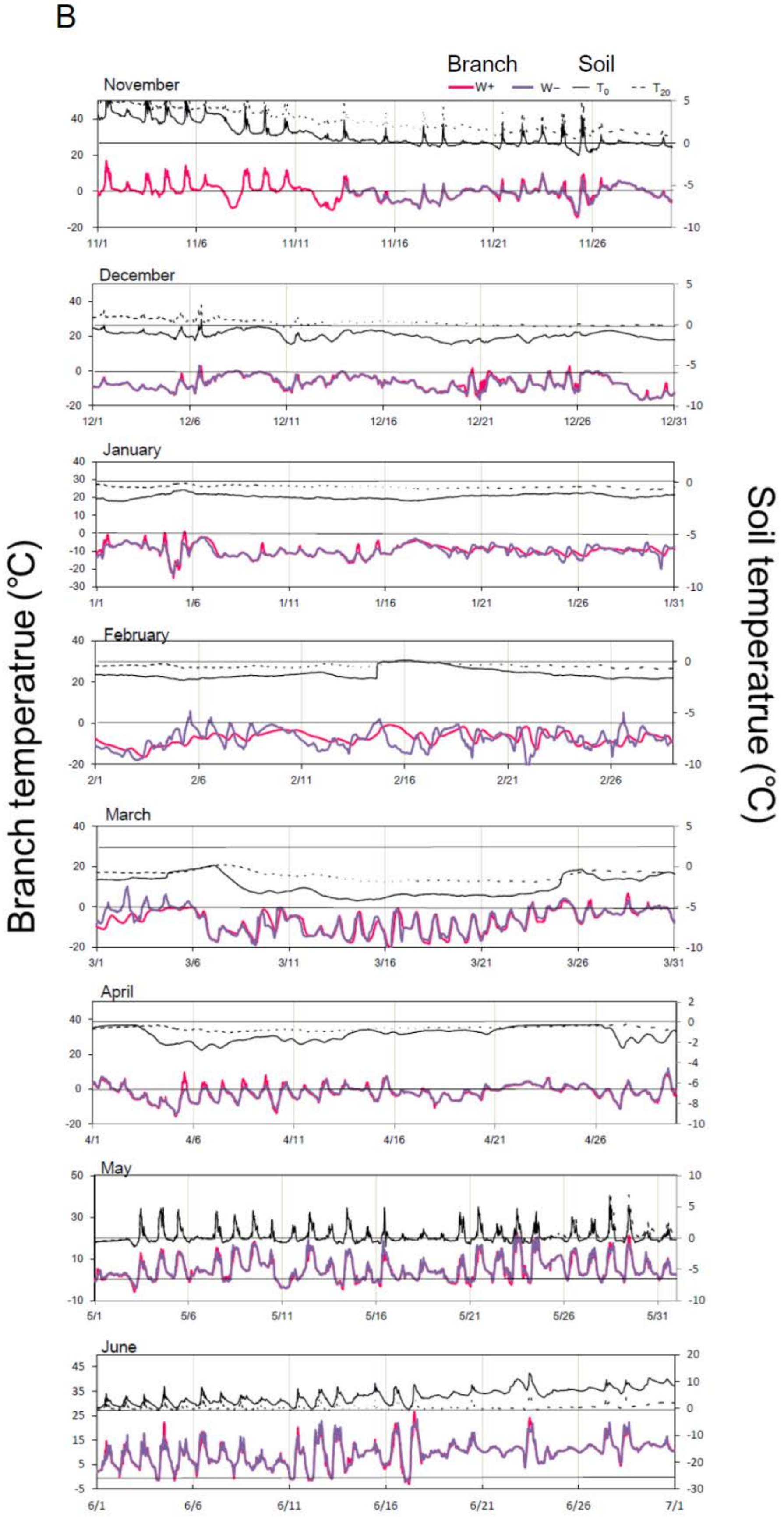

Fig. S2 Detailed representations of data shown in Fig. 8. (A) Variations in branch diameter (ΔD) from the W+ (bold line) and W− (fine line) sites. (B) Variation in branch (T_x) and soil (T_0 and T_{20}) temperatures. In December and January, the major pattern of diameter is a simple shrinkage in consistence with the sub-zero temperatures of the branch. In the period from February to April, freeze-thaw-cycle of the diameter change appears occasionally in consistence with freeze-thaw cycle of the branch temperatures. In the period from late March to April, simple swelling often occurs and lasted for 1 to several days after rainfall, although soil freezing persists (see Figs S3 and S4 for details). In early May, stomatal transpiration started immediately after the snow melted.

Fig. S3

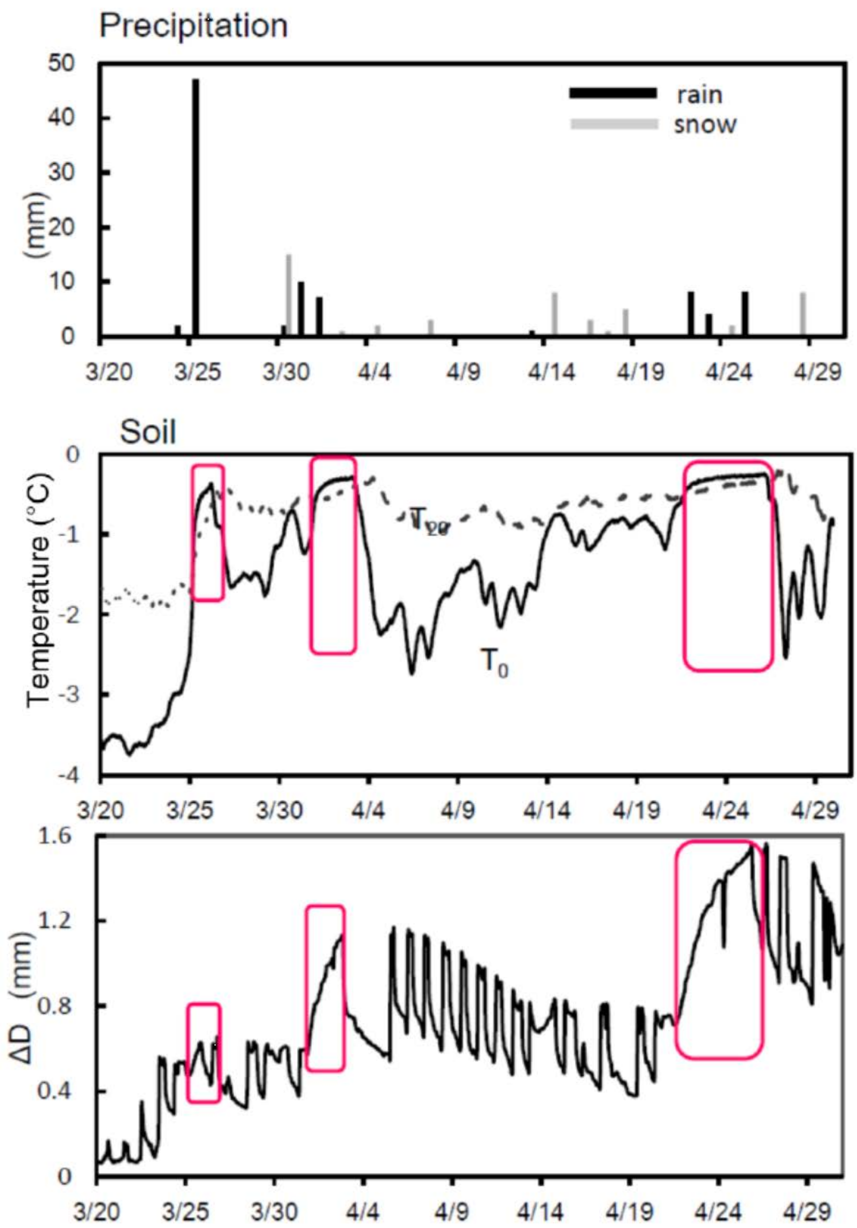

Fig. S3 Variation in precipitation, soil temperature (T_0 and T_{20}), and the variation in branch diameter (ΔD) from March 20 to April 29 at the W+ site. Data from branch diameter are the same as those shown in Fig.S2. Red rectangles delineate the rehydration period. Precipitation

data was obtained from the Japan Meteorological Agency; data were recorded at Shirakabako (1,440 m above sea level), which is 9 km north of the study site. In the precipitation graph, black bars indicate rain and grey bars indicate snow; the form of precipitation was determined based on whether the air temperature at the study site was above or below 0°C.

Fig. S4

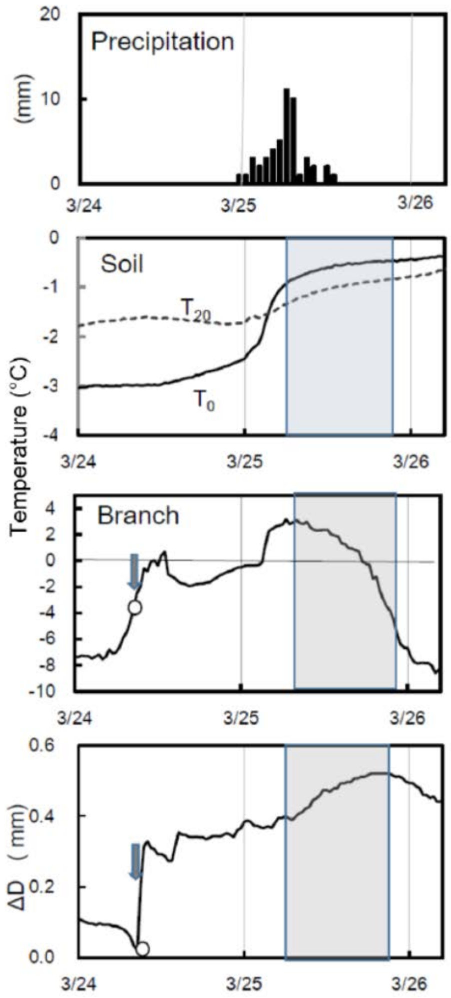

Fig. S4 The rehydration process of the bark reservoir at the first rain in early-spring (March 24–26, as shown in Fig.S2) showing precipitation, the corresponding soil temperature (T_0 and aT_{20}), branch temperature (T_x), and the variation in branch diameter (ΔD). Grey arrows indicate thaw-induced expansion, which occurred when branch temperature increased above −4°C, even if soil was frozen. Grey shading delineates the rehydration phase and the subsequent diameter

expansion. Expansion of bark due to rehydration started when T_{20} increased above −1.2°C and ceased when branch temperature decreased below −6°C, even if the soil remained melted.
